# Supplementary material for: Mechanisms governing the pioneering and redistribution capabilities of the non-classical pioneer PU.1
Source: Nat Commun. 2020 Jan 21;11:402. doi: 10.1038/s41467-019-13960-2 (PMC6972792; doi:10.1038/s41467-019-13960-2)
Supplement: Supplementary file 7 — Source data [file 41467_2019_13960_MOESM7_ESM.zip › Source_Data/FigureS2/FigureS2B_MotifScanOutput/KmeansCluster5/homerResults/motif15.similar.html]

motif15

## Information for motif15

C
T
G
A
G
C
T
A
T
C
A
G
C
G
T
A
C
A
T
G
T
A
G
C
C
A
G
T
A
C
T
G
C
T
A
G
A
G
C
T
A
C
G
T
T
A
C
G
A
G
C
T
A
C
G
T
  
Reverse Opposite:  

G
T
C
A
T
C
G
A
A
T
G
C
G
T
C
A
T
C
G
A
G
A
T
C
A
G
T
C
G
T
C
A
A
T
C
G
G
T
A
C
A
C
G
T
A
G
T
C
C
A
G
T
A
G
C
T
  

|  |  |
| --- | --- |
| p-value: | 1e-65 |
| log p-value: | -1.504e+02 |
| Information Content per bp: | 1.733 |
| Number of Target Sequences with motif | 149.0 |
| Percentage of Target Sequences with motif | 3.74% |
| Number of Background Sequences with motif | 203.8 |
| Percentage of Background Sequences with motif | 0.48% |
| Average Position of motif in Targets | 101.7 +/- 51.1bp |
| Average Position of motif in Background | 112.7 +/- 68.6bp |
| Strand Bias (log2 ratio + to - strand density) | -0.1 |
| Multiplicity (# of sites on avg that occur together) | 1.00 |
| Motif File: | file (matrix) reverse opposite |

### Similar de novo motifs found

|  |  |  |  |  |  |  |  |
| --- | --- | --- | --- | --- | --- | --- | --- |
| Rank | Match Score | Redundant Motif | P-value | log P-value | % of Targets | % of Background | Motif file |
| 1 | 0.772 | G T A C G T C A C G T A A T G C A G T C C T G A A C T G G T C A A G C T T G A C | 1e-41 | -94.597711 | 3.32% | 0.67% | motif file (matrix) |
